# Use of Digital Peer Support for Employee Well-Being: Retrospective Analysis Across Five Large Employers

**DOI:** 10.2196/90431

**Published:** 2026-04-07

**Authors:** Farbod Sedaghati, Anya Stetsenko, Ilayda Ozsan McMillan, Harpreet Nagra, Zara Dana

**Affiliations:** 1 Supportiv Berkeley, CA United States

**Keywords:** digital peer support, employee benefits, employee wellness, workforce well-being, employee assistance programs

## Abstract

**Background:**

Anonymous, 24/7 digital peer support (DPS) offers a scalable solution to support employees’ emotional well-being. Understanding sociobehavioral factors, such as timing of engagement and the impact of shared resources, can help employers and employee assistance programs (EAPs) integrate digital tools to better support workforce well-being.

**Objective:**

This study examines (1) outcomes via overall sentiment changes among DPS users; (2) sociobehavioral differences between employees who accessed real-time, anonymous DPS during versus outside business hours; (3) differences in sentiment outcomes based on time of use; and (4) the impact of in-session resource sharing on sentiment improvement.

**Methods:**

Using OpenAI’s large language model (LLM) GPT-4o-mini with a few-shot learning approach, 24,818 anonymous chat conversations from 13,879 employees at 5 large employers were evaluated for subclinical sentiment variables, including loneliness, sadness, stress, anxiety, depression, despair, helplessness, and optimism.

**Results:**

Distinct activity patterns were observed between employees during and outside business hours, with a median user age of 36 years. During business hours, employees reported higher baseline stress (Δ1.6%), whereas outside business hours, baseline depression (Δ1.7%) and loneliness (Δ1.2%) were higher. After DPS use, LLM-derived negative sentiment scores decreased (loneliness 46% reduced, sadness 45%, stress 46%, anxiety ~39%, depression ~40%, despair ~40%, and helplessness ~38%), and optimism increased (~77%). Outside business hours, users engaged more (~68% of all sessions), remained in sessions 19% longer, and discussed 13% more topics, whereas users during business hours reported greater improvements in depression (Δ2.3%), helplessness (Δ1.9%), and loneliness (Δ0.5%). Resource sharing was associated with greater improvements in loneliness (Δ2.9%), stress (Δ1.3%), anxiety (Δ1.9%), depression (Δ7.3%), despair (Δ3.0%), helplessness (Δ2.8%), and optimism (Δ8.8%), but not sadness.

**Conclusions:**

DPS complements employers’ EAPs by addressing employee engagement gaps, reducing barriers to mental health care, and promoting emotional well-being among the workforce.

## Introduction

### Background

Workplace emotional well-being is a pressing concern, as increasing numbers of employees suffer from burnout and chronic stress [1,2]. These issues lead to additional workplace problems, including absenteeism, reduced productivity, and mental health concerns [3]. Current mental health tools and employee assistance programs (EAPs) have expanded significantly in scope, evolving from a focus on mental health and substance use to include broader aspects of emotional well-being, such as family health and workplace stress [4]. However, the effectiveness of EAPs has been mixed. A 2017 systematic review concluded that EAPs benefit functioning and presenteeism but have mixed effects on absenteeism and engagement [5]. Due to the continued shortage of mental health workers, novel strategies are necessary to meet the growing demand for employee emotional support [6].

The integration of digital technology has enabled more immediate, cost-effective, and accessible forms of employee support, such as digital peer support (DPS), to be incorporated into traditional counseling-focused EAP infrastructures [7]. DPS offers an innovative approach to complement traditional EAPs, providing support on demand, in real time, by peers with shared experiences rather than clinicians, thereby promoting social connectedness, significant sentiment improvements, and emotional regulation [7,8]. However, how employees engage with DPS has yet to be studied.

### Sociobehavioral Factors

Demographic variables such as age, gender, culture, technology use, and internet access significantly affect the engagement and effectiveness of digital interventions [9-12]. Barriers such as limited technological proficiency, especially among older adults or individuals with low digital abilities, may reduce user engagement and limit program success [13]. Prior research indicates that women are more likely than men to seek mental health support and use digital mental health tools [14-17]. These differences highlight the importance of tailoring support strategies to the needs of different groups. While mobile apps and chatbots using cognitive behavioral techniques are effective in reducing symptoms of depression and anxiety, evidence shows that digital tools are more effective when supplemented with human interaction, whether through peer support or moderated participation [9].

Additionally, promoting awareness of and trust in EAP resources through culturally sensitive education has been linked to enhanced utilization among diverse employee populations [13,18]. Addressing these sociobehavioral challenges through user-centered design, sensitive outreach, and anonymous technology can improve accessibility and inclusivity across demographic groups [13,19].

### Digital Peer Support

Research shows that DPS improves emotional well-being, self-efficacy, and social connection and empathy, all of which contribute to positive organizational outcomes [14]. DPS has emerged as a critical modality for delivering emotional health and wellness services through technological platforms [7,20]. It encompasses both synchronous and asynchronous support delivered via chat, forums, video, mobile apps, and other digital communication tools, allowing users to flexibly seek mental health support [20,21]. It offers higher accessibility than more traditional services, as most individuals own a smartphone, including those from lower socioeconomic backgrounds or with serious mental illness [22-24]. Additionally, DPS has fewer barriers than in-person services, which may involve scheduling, transportation, mobility, or discomfort with face-to-face interactions [19,25], as well as tele- or mobile clinical mental health services that may require insurance, appointments, and delays due to formal clinical assessments before care can begin. As a result, DPS facilitates broader participation, particularly among populations that are underserved or less likely to engage in typical care [19,25].

In addition to accessibility, DPS services can be available 24/7, allowing continuity of care and reinforcing self-efficacy and emotional regulation, thereby filling temporal gaps left by typical mental health services [7]. Peer-delivered digital interventions have been shown to improve both psychiatric and medical self-management by empowering individuals to actively participate in their care within a supportive, nonclinical environment [20,21,25]. Reviews of DPS programs, including those for public safety workers, highlight reductions in stress and trauma [26-28], while workplace-based engagement is associated with increased belonging, accountability, and a sense of community [20,21,25]. DPS is also unique in its ability to integrate lived experience into its design, development, and delivery [21,29,30]. The involvement of peers and their lived experiences increases the relevance and acceptability of interventions [31].

The timing of user engagement is affected by a variety of sociobehavioral factors. Users who seek DPS after work hours may experience heightened levels of emotional distress, loneliness, insomnia, or anxiety, and may engage in more spontaneous or emotionally charged exchanges, whereas interactions during business hours tend to be more structured [7,20,32]. Additional barriers such as privacy concerns, stigma, and work-hour constraints can also reduce engagement during regular business hours, highlighting the need for after-hours availability of support [18,33]. Engagement patterns are also affected by demographic variables such as age, gender, employment status, education level, and housing conditions [34,35]. Preferences for specific technologies, such as text-based versus visual platforms, may vary depending on users’ mental health conditions and comfort levels [36]. For instance, some individuals may avoid video-based support and prefer text-only interactions, seeking privacy, cultural alignment, or emotional safety [37].

DPS has also demonstrated measurable improvements in emotional well-being. Services report rapid reductions in sadness (57.5%) and loneliness (55.0%), with sentiment changes emerging within minutes of engagement [8]. These benefits may be understood through relational frameworks such as social penetration theory, which suggests that trust and intimacy in relationships deepen over time, especially when individuals feel secure in their anonymity and in control over self-presentation [38]. These dynamics are often heightened during after-hours use, when users may feel more emotionally vulnerable and open, forming meaningful peer connections [32,38]. Taken together, these findings point to the critical role of timing, sociobehavioral context, and technological preferences in shaping DPS engagement.

### Role of Resource Sharing in DPS

A central mechanism of DPS effectiveness is resource sharing, which promotes mental health literacy, reduces stigma, and encourages help-seeking behaviors [27,28]. Sharing success stories and testimonials has been shown to increase employee engagement within EAPs, supporting a workplace culture that values mental health and well-being [27]. Workplace-related resource sharing also enhances coping, particularly in managing remote work, and reduces isolation [26-28]. DPS also offers timely, subclinical support during a shortage of behavioral health clinicians, helping users access resources when needed without waitlists [6,27]. Access to tailored resources may be especially valuable for underserved or marginalized populations, helping to reduce equity gaps in mental health care [13].

This study examines how sociobehavioral factors, such as the timing of support access, levels of perceived emotional distress, and in-session resource sharing, shape employee engagement and sentiment outcomes within a DPS environment. By analyzing user data across 5 large employers, we aim to identify key trends that can inform the design and implementation of digital emotional support services within workplace well-being programs.

### Study Objectives

This study examines how timing and in-session support influence sentiment outcomes within an anonymous, synchronous DPS service, Supportiv. Specifically, we investigate (1) overall sentiment changes across users of DPS; (2) sociobehavioral differences between users who accessed DPS during versus outside business hours; (3) differences in sentiment outcomes based on the timing of service use (during vs outside business hours); and (4) the impact of targeted in-session resources on users’ sentiment changes.

## Methods

### Supportiv DPS Service Model

Supportiv is an anonymous, synchronous, small-group, live-chat DPS service that is available on demand, 24/7. Employees can access the service through links from their employer portals, health plans, EAPs, or related channels. This study analyzes data from employees whose organizations offered Supportiv in addition to their existing EAP programs.

Users voluntarily access Supportiv’s anonymous peer-to-peer support service through an informed self-selection process. Engagement begins with a user-initiated response to the free-text prompt, “What’s your struggle?” Before accessing services, individuals are required to review and actively consent to the Terms of Service and Privacy Policy, which include consent for the use of their inherently anonymous data—anonymous from the point of collection—for research and service improvement purposes. No personally identifiable information, personal health information, or Health Insurance Portability and Accountability Act (HIPAA)–protected health information is collected.

To reduce the risk of indirect user identification through free-text content, all transcripts were audited using automated methods followed by manual processing before analysis. Potential personal identifiers, including names, locations, or contact information, as well as contextual details that could allow for identification, were omitted from the text. Data were stored and analyzed in deidentified form, and access was limited to authorized personnel. All data handling complies with General Data Protection Regulation (GDPR) and California Consumer Privacy Act (CCPA) requirements, including lawfulness, fairness, transparency, data minimization, purpose limitation, and integrity and confidentiality.

The platform is designed according to data protection by design and by default principles. To mitigate the risk of indirect identification in free-text content, all chats are continuously processed through automated filtering systems that remove explicit identifiers such as names, contact information, social media handles, websites, and physical addresses. In addition, trained human moderators review chats in real time and remove any remaining potentially identifying or narrowing contextual information as needed. These safeguards are applied universally across all chats as part of routine platform operations, not solely for research purposes. Access to research data is restricted to authorized personnel. Together, these safeguards reduce the risk of direct or indirect identification and ensure compliance with applicable privacy regulations.

Supportiv uses artificial intelligence (AI)–driven natural language processing (NLP) to match users with other concurrent users experiencing similar life challenges, forming small, synchronous groups of no more than 5 peers. Each group is facilitated in real time by a trained subclinical human moderator. Users are automatically placed into a chat session that may include other participants and the moderator. Moderators facilitate peer-to-peer discussion, maintain psychological safety by enforcing community guidelines, and share contextually relevant, useful resources identified through AI to match users’ expressed needs. Moderators also guide collaborative problem-solving and, in cases of crisis, promptly refer users to professional crisis services, ensuring comprehensive support.

### Study Design and Participant Enrollment

During the study period, 40,431 total chat sessions were recorded between January 2023 and December 2024. This retrospective observational study analyzed 24,818 anonymous, live user chat sessions from 13,879 employees after excluding sessions with fewer than 3 messages. Data were securely stored in the Supportiv database. Users consisted of employees from 5 large companies that implemented Supportiv’s digital peer-to-peer support service to complement their existing EAPs. The main objective was to evaluate the service’s impact on employee well-being through sentiment outcomes derived from chat session transcript analysis. The primary outcomes were within-session sentiment changes across 8 key dimensions: loneliness, sadness, stress, anxiety, depression, despair, helplessness, and optimism. Secondary descriptive outcomes included engagement metrics, use patterns, and resource-sharing outcomes. In addition to anonymous chat content, limited metadata, such as user location at the city level, derived from nonidentifying technical data, were available to support contextual analysis and geographic segmentation. No personally identifiable information or personal health information was collected.

### Ethical Considerations

As this study used a large language model (LLM) to identify sentiment scores associated with user language in DPS live chats, several ethical considerations must be addressed [39]. Concerns related to privacy are mitigated by the use of anonymous text data and the absence of user data collection. Additionally, sentiment scores were used to assess within-session emotional benefits rather than to make clinical diagnoses. All chats were facilitated by trained subclinical human moderators to support accurate sentiment interpretation and constructive DPS sessions. This retrospective study utilized operational data and did not involve direct human participant research. It was determined to be exempt from human participant research review by the Alpha Institutional Review Board (protocol IRBEMP042025).

### Conversation Setting

Each participant was matched to a chat within approximately 30 seconds of responding in free text to a single prompt: “What’s your struggle?” This response, referred to as the “user-chat-session struggle,” determined whether the user was placed into a live, ongoing small-group chat discussion on the same topic or into a 1-on-1 session with a moderator, to which others with similar struggles could later be added. Each chat could include up to 5 users and a trained subclinical professional moderator.

Moderators facilitated discussions using motivational interviewing–based OARS (open-ended questions, affirmations, reflective listening, and summaries) techniques, including open-ended questions, affirmations, reflective statements, summarizations, and structured information sharing [40]. These strategies supported conversation flow, encouraged engagement, and promoted collaborative problem-solving. To maintain continuity, moderators provided a summary for new participants joining mid-session. Moderators played a key role not only in guiding discussions and fostering a psychologically safe environment but also in reviewing AI-recommended resources, such as practical articles and informational materials tailored to users’ expressed needs, to ensure contextual sensitivity before sharing them with users.

### Sentiment Analysis

Each user chat was analyzed independently, even if the same user participated in multiple sessions. These individual sessions were referred to as “user-chat sessions.” Although some users returned for multiple sessions, the service’s anonymous nature required that each chat experience be analyzed independently, without linking sessions to the same individual. This approach ensured that each interaction was assessed in isolation, providing a clearer and more user-focused analysis of sentiment changes per intervention.

To analyze the target sentiments (loneliness, sadness, stress, anxiety, depression, despair, helplessness, and optimism), OpenAI’s LLM model, GPT-4o-mini, was leveraged using a few-shot learning technique. This method involves presenting the model with a limited number of labeled examples within the prompt to guide its outputs [41]. Previous research has demonstrated that this approach matches or exceeds the performance of state-of-the-art models [8]. Despite agreement with expert human ratings established in previous work, the LLM-derived sentiment scores should not be regarded as psychometric instruments for clinical diagnoses. These scores are indicators of expressed emotional content rather than measures of clinical symptom severity.

A user session was defined by the initial “user-chat-session struggle,” consisting of a brief free-text response of up to 300 characters, along with all subsequent messages exchanged during the chat. When a user joined a new chat session at a later time, they submitted a new struggle prompt, initiating a separate and independent session that was analyzed separately. Sentiment changes were tracked for each user within a session. Every message was assessed for sentiment intensity on a 1-10 scale, where a score of “1” indicated low intensity, “5” indicated moderate intensity, and “10” indicated high intensity for the relevant sentiment. This scale provided sufficient granularity to detect subtle variations while maintaining interpretability. The use of sentiment scoring with this model has been previously validated and reported in a prior study [8] (Multimedia Appendix 1), demonstrating strong agreement with expert human assessments. The model’s performance is continuously monitored for false positives and false negatives, and flagged misclassifications are reviewed to iteratively fine-tune the model. Importance sampling is employed to prioritize messages with high uncertainty and those near the model’s decision boundary. This strategy allows human experts to review and further refine the model’s detection of language that may involve linguistic nuances, such as indirect, metaphorical, or sarcastic expressions.

Examples illustrating scores of 1, 5, and 10 are provided in Multimedia Appendix 1. Messages not suitable for sentiment analysis, such as clarifying or irrelevant statements, were excluded (eg, “Or a skill,” “What do you mean,” “At what time?”). To reduce misclassification, the model was instructed to refrain from assigning scores when insufficient information was available to infer sentiment content.

Due to the DPS service’s voluntary live chat model, validated multiitem self-report instruments such as the 9-item Patient Health Questionnaire (PHQ-9), 7-item Generalized Anxiety Disorder (GAD-7), or 10-item Perceived Stress Scale (PSS-10) were not administered to users. Although ecological momentary assessments and visual analog scales can support in-the-moment measurement, they are typically used in structured research protocols and rely on repeated prompts and user incentives. By contrast, peer support environments prioritize user autonomy and natural conversational flow; therefore, repeated prompts may be disruptive. Additionally, many symptom instruments rely on self-report, which is prone to bias, social pressure, reduced introspection, distortion, and response fatigue. In this context, LLM-assigned sentiment scores were used as conversationally embedded measures rather than validated instruments. These scores were based on the language used by participants. Our previous study demonstrated that this approach is comparable to state-of-the-art models [8]. Additionally, rather than indicating diagnostic severity, sentiment scores were used to assess within-session changes. This is consistent with prior research using NLP to evaluate digital interventions at scale [42-44]. These scores were not intended to represent diagnostic severity; instead, they provide scalable indicators of expressed emotional content to assess within-session sentiment changes. Future validation work may assess how NLP-derived indicators align with standardized measures (such as PHQ-9, GAD-7, and PSS-10).

### Inclusion and Exclusion Criteria

To ensure a meaningful assessment of sentiment trends, specific criteria were applied when selecting chat sessions for analysis. Only conversations in which users engaged beyond a single message were included, as single-message interactions did not provide sufficient data to track sentiment changes over time.

For each user chat session, 8 emotions were screened to determine whether the session contained an interpretable sentiment change. To ensure the analysis focused on sessions with meaningful sentiment expression, a threshold score of 5 was established. For negative emotions (loneliness, sadness, stress, anxiety, depression, helplessness, or despair), sessions were included if the initial score was greater than 5, whereas for the positive emotion (optimism), a score below 5 was required. The initial emotional state was defined using the highest sentiment score from the user’s first 3 messages, recognizing that individuals often begin conversations with brief or casual exchanges before revealing more meaningful emotional content and underlying struggles. This approach captured users who eventually expressed their emotional concerns while filtering out sessions lacking sufficient sentiment depth for analysis.

### Sentiment Analysis Across All User Chat Conversations

To evaluate sentiment states over time across user chat sessions, the sentiment data were standardized to allow comparison despite variation in conversation duration. Each conversation was normalized to a relative scale (from 0 to 1) using bins of size 0.01. If a message with a sentiment score was present within a bin, the score was assigned to that bin. This normalization process allowed sentiment trends to be compared across conversations.

After aggregating the binned data for each sentiment, a linear trend line was fitted. The slope of this line indicated the direction and magnitude of sentiment change, reflecting the service’s effectiveness in supporting well-being. The goodness of fit was evaluated using the coefficient of determination (*R*^2^). Finally, CIs were calculated to represent the standard error of the data, indicating variability around the mean for each conversation duration bin.

### Propensity Matching

Propensity matching was used to create comparable cohorts for each research question. Users within the cohorts were matched by training a logistic regression classifier using the following features: user-chat-session topic, device type, user country, number of users in the chat, conversation duration, engagement rate (number of characters typed by the user per unit of conversation time), and initial sentiment score. This matching ensured a high level of alignment between users in the 2 cohorts at the start of the chat by incorporating both demographic and behavioral metrics.

To ensure meaningful matching, conversation duration and engagement rate were transformed into categorical bins using predefined ranges. For conversation duration, conversations in each cohort were divided into the following bins based on length (in minutes): “0-5,” “5-10,” “10-20,” “20-45,” “45-60,” and “>60.” Similarly, for engagement rate (measured as the number of characters typed by the primary user per unit of time), bins were created to categorize conversations based on characters typed per unit time: “0-10,” “10-20,” “20-30,” “30-40,” “40-50,” and “>50.”

The logistic regression model produced a propensity score for each user chat session, representing the likelihood of assignment to one cohort. We used 25% of the SD of all propensity scores as the caliper (radius of search) for matching. For each user chat in 1 cohort, the closest match in the other cohort was identified using a k-nearest neighbor classifier within the caliper. Propensity matching was conducted separately for each trend analyzed.

The topics for user chat struggles were categorized into the following themes (alphabetically): anxiety, caregiving/parenting, depression, financial/social determinants of health, identity, loneliness, mental health conditions, physical health/medical, relationship issues, social connection and belonging, stress, suicide/self-harm, trauma, and work/productivity/burnout. These categories were identified through manual inspection of chats to determine common themes across the population.

### Analyzing Employee User Experiences

To gain a comprehensive understanding of employee users and characterize their engagement with the service, several key components were analyzed. First, although the service is anonymous, users may voluntarily provide demographic information and optional feedback at the end of a chat session. Analysis of these data offers valuable insights into the study population.

The primary target group consisted of employees from 5 large employers across varied industry sectors, including retail, media, and health care. As these organizations differed in job roles and workforce structure, engagement patterns were analyzed across heterogeneous contexts, enhancing ecological validity and reducing bias. User engagement patterns were examined throughout the day to determine whether usage varied between business and nonbusiness hours. For this analysis, business hours were defined as Monday through Friday from 8:00 AM to 5:00 PM local time, while all other periods, including evenings, nights, and weekends, were categorized as nonbusiness hours.

A core advantage of the Supportiv service is its ability to deliver highly personalized resources tailored to users’ stated struggles and needs. Supportiv enables moderators to share coping tools, self-help materials, and other resources, as well as links to relevant employee benefits or community resources, specifically tailored to a user’s needs based on context. These resources include various formats such as articles, videos, audio files, tools, worksheets, exercises, community resources, and client-specific materials. Resource sharing is driven by a 2-stage AI recommendation system. The AI model retrieves relevant items that match users’ expressed needs from a resource library and then ranks the retrieved items to ensure that the most appropriate and valuable options are prioritized. Moderators review and select from this AI-curated list before sharing resources with users. The analysis examined the types of resources recommended by AI and shared by moderators, as well as how users interacted with them.

Lastly, although all target users were employees utilizing Supportiv through sponsored links (via their EAPs, employee benefits portals, or other employer-specific URLs), moderators may refer employees to additional relevant EAP services based on their expressed needs. These EAP-related metrics were analyzed to better understand how employees used integrated support services and how DPS functioned within the organizational benefits system.

### Sentiment Trends

#### Variables Analyzed

Two primary variables were independently analyzed for their influence on user sentiment trends: (1) time of engagement and (2) in-session resource sharing.

#### Time of Engagement

We hypothesized that users engaging with the service outside business hours would exhibit different sentiment outcomes compared to those using it during business hours. To evaluate this, chat sessions initiated during business hours were compared with those started outside business hours, with the user’s time zone used to convert the system’s default time to local time for cohort classification.

#### Resource Sharing

We also hypothesized that receiving a shared resource would promote positive sentiment trends, regardless of the type of resource or the extent of user interaction with it.

For both variables, sentiment evolution throughout each chat session was analyzed, and propensity matching was employed to create 2 comparable cohorts for each target variable. This matching procedure ensured a fair comparison between groups to effectively assess how the effects of time of engagement and in-session resource sharing shape sentiment outcomes.

### Statistical Analysis

Statistical analyses were conducted to (1) compare differences between groups for specific features, (2) evaluate the significance of predicted sentiment trends, and (3) compare sentiment improvement metrics (slopes) between target cohorts. Statistical significance was defined as *P*<.05, and all tests were 2-tailed. Independent contractors were engaged to develop the analysis plan and conduct statistical analyses to minimize commercial bias. Effect sizes for pairwise comparisons were computed using Cohen *d*.

All analyses were performed using Python 3.9.6 (Python Foundation) with the following libraries: pandas 2.3.3, NumPy 2.0.2, SciPy 1.13.1, scikit-learn 1.6.1, statsmodels 0.14.6, matplotlib 3.9.4, and seaborn 0.13.2.

### Topic Modeling

We utilized a topic-modeling framework based on LLM-driven categorization to assign topics and subtopics to text data. After reviewing the conversations, a list of topics was manually defined. All texts were then processed using GPT-4o-mini with a structured prompt to categorize them into the predefined categories. Classification accuracy was manually verified, and, when necessary, categories were corrected manually.

## Results

### Chat Statistics

A total of 40,431 chat sessions were recorded during the study period. Sessions containing only 1 or 2 messages were excluded, as they lacked sufficient context to evaluate sentiment trends or changes over time. After applying these criteria, 24,818 chat sessions from 13,879 employees with at least three messages were included in the analysis.

### User Reported Struggle Topics

Users often reported more than 1 concern during their chat sessions. The most common struggles were relationship issues (7531/24,818, 30.34%), trauma (2663/24,818, 10.73%), workplace concerns (2650/24,818, 10.68%), stress (2074/24,818, 8.36%), loneliness (1757/24,818, 7.08%), depression (1570/24,818, 6.33%), and financial/social determinants of health (1565/24,818, 6.31%). Additional struggles included support system challenges (1160/24,818, 4.67%), anxiety (1098/24,818, 4.42%), mental health conditions (723/24,818, 2.91%), identity concerns (638/24,818, 2.57%), caregiving/parenting (617/24,818, 2.49%), physical health/medical concerns (399/24,818, 1.61%), and suicide/self-harm (373/24,818, 1.50%).

### User Activity

On average, users spent 37.97 (SD 35.51) minutes in peer group chats. The average number of messages sent by the primary user was 24.42 (SD 29.45). Variation in chat duration, as reflected by time spent and number of messages, indicates that the analysis includes both users who engaged in extended conversations and those who participated for shorter periods but met the minimum inclusion criteria. To better capture user participation intensity, a metric termed “characters per chat duration” was calculated, with an average of 70.65 (SD 1394.24) characters per minute. The high SD in this metric may be attributed to differences in users’ communication styles. While some users type long, paragraph-style messages, others send multiple short messages consisting of only a few words at a time.

**Figure 1:**
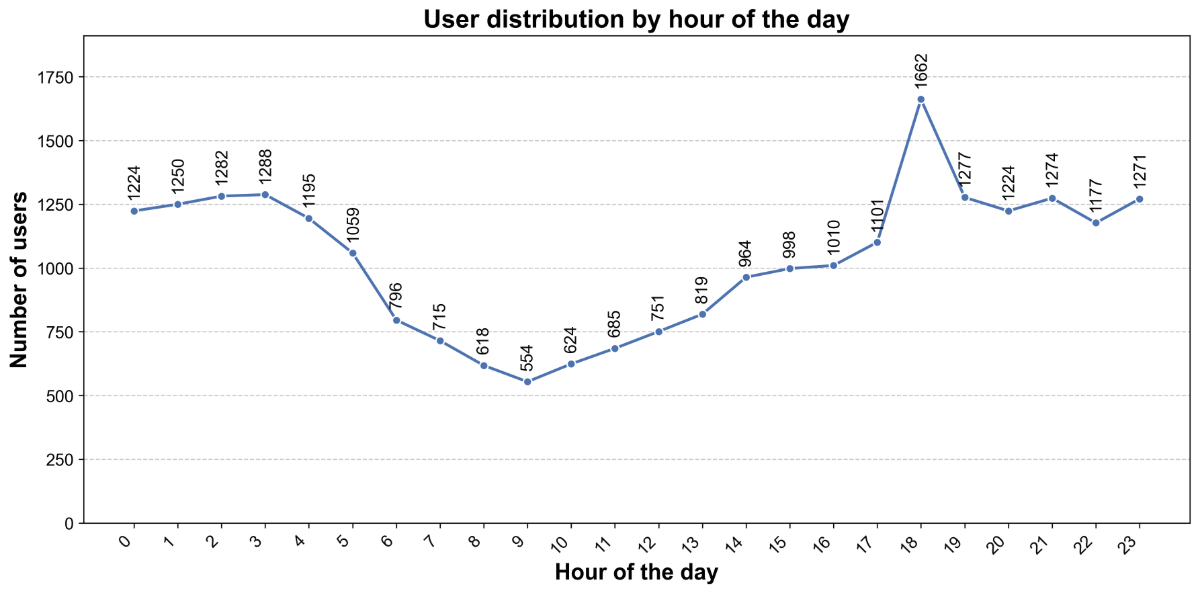
Distribution of conversation start times based on local time over a 24-hour period. The graph shows the frequency of user chat sessions throughout the day.

Figure 1 illustrates the number of users throughout the day. User activity decreases during late-night hours, increases steadily as business hours begin, and peaks toward the end of the day before declining at night. Notably, user engagement was higher outside business hours, suggesting that employees may be more likely to use DPS during their personal time, when they are away from job responsibilities and have more space to share their thoughts and concerns without distraction.

### Study Population

#### Demographics

Employees from all global regions accessed the DPS service, with the majority of conversations (16,720/20,976, 79.71%, chats with reported location information) located in the United States. Most users accessed the service via mobile devices (19,854/24,817, 80%), followed by desktop (4467/24,817, 18%) and tablets (496/24,817, 2%). Although the service is anonymous, users have the option to voluntarily share demographic information, including age, race, and gender. Out of 24,818 anonymous chat conversations, 8647 (34.84%) users disclosed demographic information, which is a relatively high response rate compared with similar digital mental health services [45]. While demographic data were not available for all users, the information that was voluntarily provided offered valuable insights and was incorporated into the propensity matching algorithm to enhance comparative sentiment analyses among similar users.

Table 1 presents the distribution of user ages, grouped into 3-year increments. Overall, the median age of the users is 36 years, with a mean of ~45 years, indicating that DPS attracts a broad adult population. Individuals aged 37-57 generally access psychological services less frequently [46]. However, the accessibility and anonymity of Supportiv’s DPS model may make it a more viable option for this group by offering an easier point of entry to mental health support.

In conversations with users who disclosed their gender, 5738 (66.36%) identified as female, 2538 (29.35%) as male, and 371 (4.29%) as nonbinary. This distribution aligns with the literature showing that, despite using digital services more frequently [47], men are significantly less likely to seek mental health support than women [48]. The use of DPS by nonbinary users may reflect its inclusive and anonymous design, which can reduce barriers often encountered in traditional clinical settings. This inclusivity highlights the potential for DPS services to foster psychological safety.

In terms of race and ethnicity among conversations with those who provided their ethnicity/race demographic information (n=8914), 4759 (53.39%) identified as White, followed by African American (n=1469, 16.48%), Hispanic (n=1290, 14.47%), and Asian (n=810, 9.09%) users, with the remainder reporting other minority groups (n=309, 3.47%, American Indian or Alaska Native; n=171, 1.92%, Native Hawaiian or Other Pacific Islander; and n=106, 1.19%, Middle Eastern or North African). This diversity suggests that DPS services are effective in reaching historically underrepresented groups. Anonymous access and culturally neutral peer interaction may help reduce barriers to accessing support services.

**Table 1:** Distribution of users by age.^a^

User age (years)	Count, n
13-15	239
16-18	954
19-21	833
22-24	691
25-27	616
28-30	630
31-33	582
34-36	640
37-39	275
40-42	484
43-45	238
46-48	266
49-51	466
52-54	194
55-57	367
58-60	169
61-63	172
64-66	106
67-69	149
70-72	91
73-75	85
76-78	139

^a^Demographic data were obtained from users who voluntarily disclosed age information (8386/24,818, 33.79%).

#### EAP Inflows and Outflows

Tables 2 and 3 provide perspectives on the relationship between DPS and EAP utilization, reflecting users referred to DPS through their EAP programs (inflow) and those referred to EAP programs after using DPS (outflow). These sessions were initiated by employees with varying job roles from 5 large employers representing multiple industries, including retail, media, and health care. Table 2 (EAP inflows) presents the distribution of users who accessed the service through their organization’s EAP, categorized by struggle topic. This table shows how employees are reaching DPS and aims to identify the most common concerns prompting employees with existing EAP benefits to use DPS services. Of 24,818 total user chat sessions, 5482 (22.09%) were initiated through users’ EAP programs. Table 3 (EAP outflows) illustrates the distribution of users who were referred to their organization’s EAP services through DPS sessions, also categorized by struggle topic. This reflects continuity of care from DPS chat sessions—based on moderator assessment or users expressing need—to additional employer-provided services. Of 24,818 user chat sessions, 2720 (10.96%) were referred to EAP services after DPS sessions.

Notably, the proportions of inflow and outflow vary across topics, suggesting that certain concerns may be more likely to drive employees to seek support through DPS, while others necessitate referrals to EAP resources. These insights can help refine targeted support strategies across both DPS and EAP services, ensuring that users receive effective care that bridges gaps in traditional care. For example, topics such as relationship issues, loneliness, stress, and trauma are among the most common concerns and may show higher referral rates, indicating opportunities to strengthen collaboration between DPS and EAPs for these specific issues.

**Table 2:** Employee users who accessed the Supportiv service through their organization’s EAP^a^ program referral.

Struggle topic	Count (N=24,818), n	EAP (N=5482), n (%)^b^
Anxiety	1098	363 (33.06)
Loneliness	1757	513 (29.20)
Physical health/medical	399	116 (29.07)
Suicide/self-harm	373	107 (28.69)
Trauma	2663	659 (24.75)
Relationship issues	7531	1840 (24.43)
Identity	638	140 (21.94)
Mental health conditions	723	157 (21.72)
Depression	1570	324 (20.64)
Social connection and belonging	1160	233 (20.09)
Stress	2074	387 (18.66)
Caregiving/parenting	617	87 (14.10)
Work/productivity/burnout	2650	356 (13.43)
Financial/Social Determinants of Health	1565	200 (12.78)

^a^EAP: employee assistance program.

^b^Percentages derived as follows: per struggle topic, (EAP n / Count n) × 100.

**Table 3:** Employee users who were referred back to EAP^a^ resources after participating in a DPS^b^ chat session.

Struggle topic	Count (N=24,818), n	EAP (N=2720), n (%)^c^
Anxiety	1098	207 (18.85)
Depression	1570	252 (16.05)
Caregiving/parenting	617	83 (13.45)
Mental health conditions	723	97 (13.42)
Suicide/self-harm	373	50 (13.40)
Trauma	2663	342 (12.84)
Loneliness	1757	210 (11.95)
Stress	2074	235 (11.33)
Social connection and belonging	1160	120 (10.34)
Relationship issues	7531	764 (10.14)
Physical health/medical	399	40 (10.03)
Identity	638	43 (6.74)
Financial/Social Determinants of Health	1565	105 (6.71)
Work/productivity/burnout	2650	172 (6.49)

^a^EAP: employee assistance program.

^b^DPS: digital peer support.

^c^Percentages derived as follows: per struggle topic, (EAP n / Count n) × 100.

#### Shared Resources

A key feature of the Supportiv service is its ability to share contextually relevant, tailored resources with users seeking emotional support, enhancing the user experience and the effectiveness of the DPS service on sentiment outcomes. In total, 33,632 resources were shared across 24,818 chat sessions, averaging 1.36 resources per session, indicating that at least one relevant resource was shared per session. Among these resources, 16,157 were articles, 8813 were videos, 5269 were employer-specific benefits, 2581 were worksheets, 810 were inspirational quotes, and 2 were audio files. Table 4 shows the distribution of resource types across various struggle topics, with an average of 2402 resources shared per topic.

**Table 4:** Distribution of resource types shared with users across various struggle topics.

Struggle topic	Blog (N=16,157), n	Video (N=8813), n	Employee specific (N=5269), n	Worksheet (N=2581), n	Quote (N=810), n	Audio (N=2), n	Count (N=33,632), n
Relationship issues	5338	3089	1314	983	332	2	11,058
Trauma	1866	956	590	382	74	0	3868
Work/productivity/burnout	1556	749	629	170	68	0	3172
Stress	1347	736	496	269	55	0	2903
Loneliness	1174	649	332	195	80	0	2430
Depression	974	516	365	151	47	0	2053
Financial/Social Determinants of Health	871	382	442	64	27	0	1786
Social connection and belonging	651	364	227	91	35	0	1368
Anxiety	753	440	265	99	35	0	1592
Mental health conditions	453	261	154	37	15	0	920
Identity	380	217	104	47	11	0	759
Caregiving/parenting	361	219	158	45	14	0	797
Physical health/medical	219	128	118	19	8	0	492
Suicide/self-harm	214	107	75	29	9	0	434

#### User Struggles

The 5 most common struggle topics expressed by users during business hours (total chats n=7995) were relationship issues (n=2249, 28.13%), workplace concerns (n=1029, 12.87%), financial or Social Determinants of Health (n=744, 9.31%), trauma (n=734, 9.18%), and stress (n=732, 9.16%). By contrast, outside business hours (total chats n=16,823), users most frequently reported relationship issues (n=5282, 31.40%), trauma (n=1929, 11.47%), workplace concerns (n=1621, 9.64%), stress (n=1342, 7.98%), and loneliness (n=1313, 7.80%). These patterns suggest that, while some concerns are consistently reported throughout the day, workplace and financial concerns are more prevalent during business hours, when work-related stressors affect users. Outside business hours, loneliness and trauma are more commonly reported, indicating that users are more open to engaging in personal discussions during nonworking hours.

#### User Activity

Several user activity metrics were analyzed for the 2 cohorts in the business-hour study. These included conversation duration (time from chat initiation to exit), typing activity (number of typed characters per minute as an indicator of engagement), total messages and characters sent, and the number of topics discussed during the chat.

**Figure 2:**
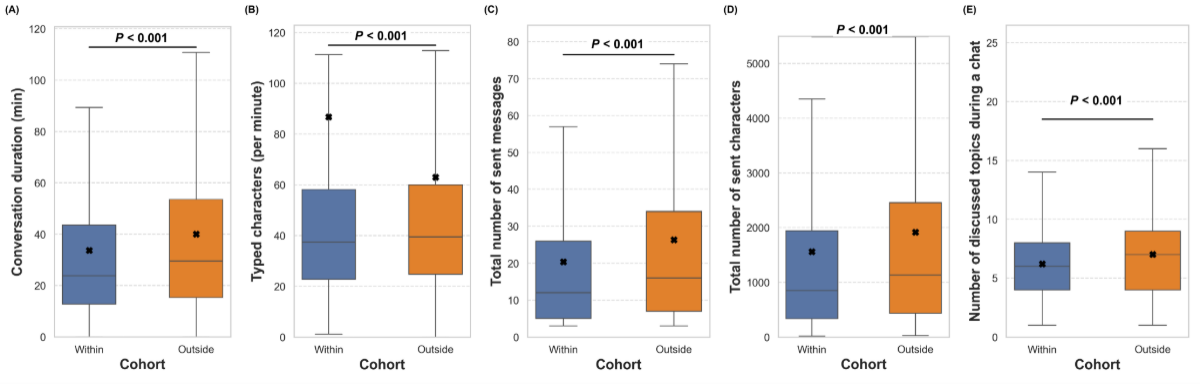
User activity in the "within" versus the "Outside" business hours groups are separated based on (A) conversation duration, (B) characters sent per minute, (C) number of sent messages, (D) number of sent characters, and (E) number of discussed topics, by the user. The value denoted by "x" is the mean.

As shown in Figure 2A, a significant difference (*P*<.001) was observed in conversation duration between user chat sessions. The mean conversation duration was 33.63 (SD 32.72) minutes for the “within-business-hours” cohort and 40.03 (SD 36.59) minutes for the “outside-business-hours” cohort, indicating that users in the “outside-business-hours” group stayed on the service 19% longer than those in the “within-business-hours” group.

User participation, measured by typing activity, also differed significantly between groups (*P*<.001; Figure 2B). During business hours, users typed an average of 86.75 (SD 2316.61) characters per minute, compared with 63.00 (SD 563.27) characters per minute outside business hours. This suggests that users during business hours typed 38% faster.

Another key engagement metric is the number of messages and characters sent. Users in the “within-business-hours” group sent an average of 20.36 (SD 25.55) messages and 1559.61 (SD 2084.01) characters, whereas those in the “outside-business-hours” group sent 26.35 (SD 30.95) messages and 1917.05 (SD 2406.78) characters, representing increases of 29% and 23%, respectively. These differences were significant between the 2 groups (both *P*<.001; Figures 2C and 2D).

Users discussed an average of 6.21 (SD 3.28) topics in chat sessions during business hours, compared with 7.01 (SD 3.53) topics outside business hours, representing a 13% increase outside business hours (*P*<.001; Figure 2E). Taken together, these findings indicate differences in user engagement patterns between the 2 cohorts. Users who used DPS outside business hours remained in chats longer, exchanged more messages, and covered more topics, suggesting that they were more likely to engage at a deeper and more personal level when work-related stressors, distractions, or time constraints were not present.

### Sentiment Outcomes and Trends

#### Within-Session Sentiment Trajectories Across DPS Engagement

Changes in users’ sentiment outcomes for loneliness, sadness, stress, anxiety, depression, despair, helplessness, and optimism were analyzed from the beginning to the end of each moderated, peer-to-peer live chat session to assess whether engaging with DPS was associated with improved sentiment scores. These trends provide an overview of the service’s general sentiment support and serve as a reference point for interpreting more targeted results in subsequent sections.

**Figure 3:**
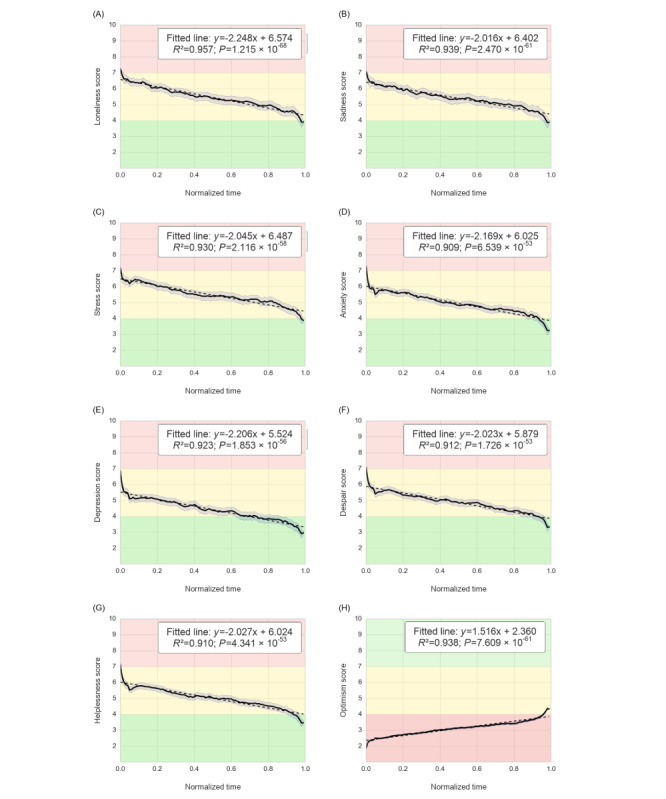
Sentiment outcomes trends throughout live chat peer-to-peer conversation progression. Sentiment changes were observed as conversations progressed. The solid black line shows the emotion score across the conversation, the dashed line represents the fitted line, and the shaded area represents the SEM. The fitted line information is provided in the box, which includes the slope (how fast the emotion is reduced per normalized conversation time), the intercept (the approximate initial emotion score), and the goodness of fit. (A–G) The green area (1-4) represents “low” levels of emotion, yellow (4-7) signifies “moderate” levels, and red (7-10) indicates “high” momentary emotion. (H) The red area represents low optimism (1-4), the yellow area represents neutral optimism (4-7), and the green area represents positive optimism (7-10).

As shown in Figure 3, the 8 time-series graphs illustrate how users’ sentiment states evolved throughout the course of the live chat sessions. Each graph displays one of the key dimensions (loneliness, sadness, stress, anxiety, depression, despair, helplessness, and optimism) with a fitted linear regression line, where the x-axis represents normalized session time and the y-axis represents the respective sentiment scores on a 10-point scale. These plots capture trends across all users, offering a holistic picture of Supportiv’s influence on sentiment change, independent of contextual factors such as session timing or receipt of a resource.

The results reveal consistently improved sentiment states across all 8 dimensions. Notably, all negative sentiments (loneliness, sadness, stress, anxiety, depression, despair, and helplessness) declined steadily over the course of a session, while the positive sentiment of optimism increased. The negative sentiments showed strong, pronounced downward trends. As not all conversations included all sentiments, the number of conversations per sentiment analysis differed. The greatest changes were observed in loneliness, which was reduced by 46% (n=11,492; *R*^2^=0.957; *P*<.001; Figure 3A); sadness, by 45% (n=10,741; *R*^2^=0.939; *P*<.001; Figure 3B); and stress, by 46% (n=13,928; *R*^2^=0.93; *P*<.001; Figure 3C). These were followed by anxiety, which was reduced by about 39% (n=9654; *R*^2^=0.909; *P*<.001; Figure 3D); depression, by about 40% (n=5585; *R*^2^=0.923; *P*<.001; Figure 3E); despair, by about 40% (n=6197; *R*^2^=0.912; *P*<.001; Figure 3F); and helplessness, by about 38% (n=7742; *R*^2^=0.910; *P*<.001; Figure 3G), highlighting the consistency of sentiment relief across users. The downward trends in negative sentiment scores suggest that users’ expressed emotional language became less indicative of negative affect over DPS chats. By contrast, optimism increased by about 77% (Figure 3H). This sustained upward trend (n=23,491; *R*^2^=0.938; *P*<.001) suggests that users not only experienced relief from distress during DPS conversations but also left sessions feeling more hopeful and supported. Notably, these changes occurred smoothly over time, with minimal noise or volatility, as shown by the narrow CIs in most graphs.

Taken together, these findings indicate that DPS use was consistently associated with improvements in within-session sentiment scores. The strength and stability of the observed trends confirm improved sentiments following DPS use. Overall, users began sessions with high-intensity negative affect and concluded within the neutral-to-positive range by the end of the sessions, illustrating that engagement with DPS is associated with favorable shifts in sentiment scores.

#### Business Hour Effect

Consistent with previous research [7], user engagement revealed that nearly two-thirds of sessions occurred outside business hours, with 16,823 chats outside business hours and 7995 during local business hours (Figure 1). Table 5 presents the initial baseline sentiment states of users who accessed the service during business hours compared with those who did so outside business hours. Baseline scores were similar for most sentiments, although a few showed differences. Users who accessed the DPS service during business hours reported slightly higher stress levels (*P*<.001), suggesting that work-related stressors may contribute to increased distress during business hours. Conversely, users who accessed DPS after business hours reported higher levels of loneliness (*P*=7.63 × 10^–3^), possibly due to increased isolation during evening and nighttime hours. No significant differences were found in anxiety (*P*=.54), despair (*P*=.23), helplessness (*P*=.58), sadness (*P*=.26), or optimism (*P*=.15) scores. These results suggest that employees may seek support with different baseline sentiment states depending on the time of engagement.

**Figure 4:**
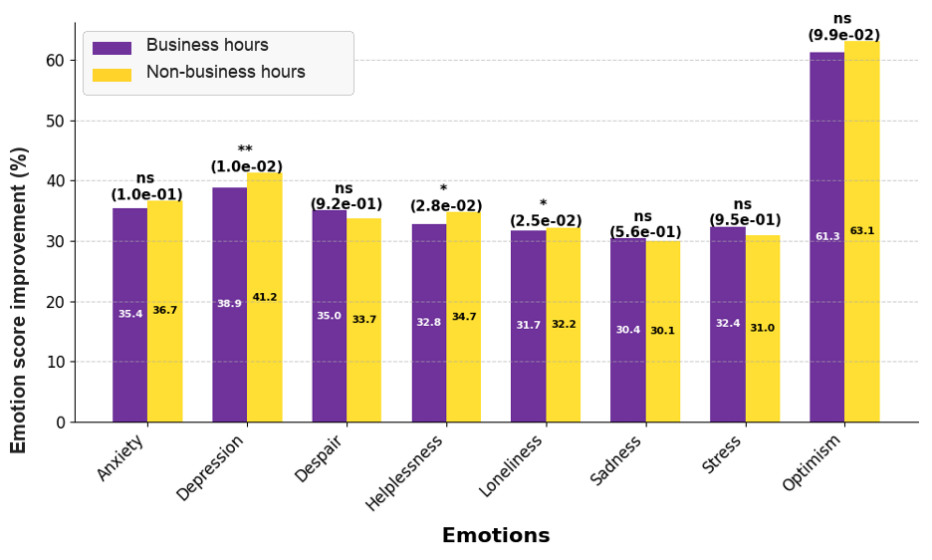
Emotion score improvements by time-of-service use: users engaged during business hours experienced greater reductions in depression, helplessness, and loneliness. No significant differences were found for anxiety, despair, sadness, stress, or optimism. The improvement percentages were calculated based on the slope of the fitted trend line. *P* values are indicated in brackets. *P<.05. **P<.01.

Figure 4 compares sentiment score improvements between users who initiated chat sessions during local business hours (within) and those who did so outside business hours (outside) across 8 sentiment domains: anxiety, depression, despair, helplessness, loneliness, sadness, stress, and optimism. Users who utilized DPS outside business hours showed significantly greater reductions in depression (41.2% vs 38.9%; *P*=1.0 × 10^–2^; *d*=0.0197), helplessness (34.7% vs 32.8%; *P*=2.8 × 10^–2^; *d*=0.0058), and loneliness (32.2% vs 31.7%; *P*=2.5 × 10^–2^; *d*=0.0166), suggesting that support outside work hours was associated with greater changes in sentiment scores, although the effect sizes were small. No statistically significant differences were observed for anxiety (*P*=.10), despair (*P*=.92), sadness (*P*=.45), stress (*P*=.62), or optimism (*P*=.10), and changes in sentiment scores were similar for users during and after business hours. These results also suggest that engagement during business hours may serve as an important source of immediate support and validation for employees, allowing them to process their thoughts and feelings while helping sustain concentration during the workday.

**Table 5:** Comparison of baseline sentiment scores between business hours and nonbusiness hours users.

Factor	Anxiety	Depression	Despair	Helplessness	Loneliness	Sadness	Stress	Optimism
Within business hours, mean (SD)	6.24 (2.40)	5.17 (2.29)	5.82 (2.31)	6.17 (2.33)	6.66 (1.99)	6.62 (1.82)	6.96 (1.85)	3.12 (1.64)
Outside business hours, mean (SD)	6.25 (2.29)	5.26 (2.16)	5.88 (2.19)	6.17 (2.27)	6.74 (2.00)	6.58 (1.83)	6.85 (1.86)	3.10 (1.65)
*P* value	.54^a^	.04^b^	.23^a^	.58^a^	7.63 × 10^–3c^	.28^a^	<.001	.15^a^

^a^Not significant.

^b^*P*<.05.

^c^*P*<.01.

#### Resource Sharing Effect

**Figure 5:**
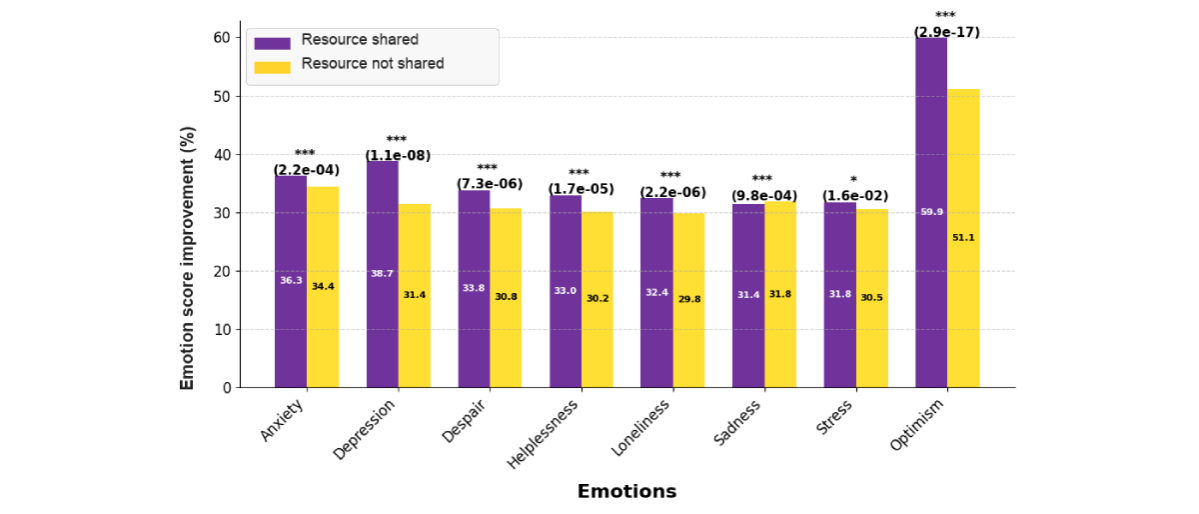
Comparison of emotion score improvements between users who received at least one resource and those who did not. Resource sharing was associated with significantly greater reductions in anxiety, depression, despair, helplessness, loneliness, and stress, and gains in optimism, while sadness slightly favored the nonreceiving group. The improvement percentages were calculated based on the slope of the fitted trend line. *P* values are indicated in brackets. *P<.05; ***P<.001.

Figure 5 compares sentiment score improvements between users who received at least one resource during their chat session (with) and those who did not (without) across 8 dimensions: anxiety, depression, despair, helplessness, loneliness, sadness, stress, and optimism. Users who received a resource showed significantly greater changes in almost all sentiment states: anxiety (36.3% vs 34.4%; *P*<.001; *d*=−0.0003), depression (38.7% vs 31.4%; *P*<.001; *d*=−0.0830), despair (33.8% vs 30.8%; *P*<.001; *d*=−0.0468), helplessness (33.0% vs 30.2%; *P*<.001; *d*=−0.0752), loneliness (32.7% vs 29.8%; *P*<.001; *d*=−0.0789), stress (31.8% vs 30.5%; *P*=1.6 × 10^–2^; *d*=−0.0342), and optimism (59.9% vs 51.1%; *P*<.001; *d*=−0.0145). Interestingly, users who did not receive a resource showed a slightly greater reduction in sadness (31.8% vs 31.4%; *P*<.001; *d*=−0.0287), although the difference was negligible. Overall, these findings highlight an association between resource sharing and improved sentiment scores, particularly in alleviating negative sentiment states and enhancing optimism. Therefore, resource provision serves as a supportive tool when incorporated into DPS services.

## Discussion

### Principal Findings

This study offers novel insights into the role of timing, in-chat behaviors, and structural barriers in shaping user engagement and sentiment outcomes in a DPS service offered as a stand-alone service or integrated into EAPs. The findings advance understanding of when and how employees use DPS services, how these interactions guide sentiment trajectories, and which factors affect access to mental health support.

### Barriers to EAP Utilization

Despite the potential of DPS to fill critical gaps in care, broader EAP engagement remains limited due to persistent systemic barriers. The most pressing among these is a widespread lack of awareness about the scope and availability of EAP services, leading to underutilization even in organizations with robust offerings [49]. Research shows that, without sustained promotional efforts, EAP usage rates remain below 10%, while programs with active education and engagement campaigns have reported utilization rates closer to 30% [50,51]. Additionally, internalized stigma may deter individuals from seeking help [52]. Employees may interpret help-seeking as a signal of personal failure or professional inadequacy, especially in high-performance cultures. National surveys indicate declining employee comfort with employer-sponsored mental health services, with only 52% of employees reporting comfort accessing EAP resources in 2022, down from 64% in 2021 and 67% in 2020 [53]. Addressing these gaps requires shifts at the organizational level, including visible leadership endorsement of mental health resources, normalization of mental health care, and integration of DPS into wellness initiatives. These strategies can help develop EAPs from crisis tools into preventive support systems and increase their perceived legitimacy [54].

### Sociobehavioral Factors Influencing Use

Beyond timing, additional sociobehavioral factors also influenced engagement with DPS. Although many users identified as female and reported White ethnicity, as expected for mental health services [55], DPS attracted a larger proportion of male and Black, Indigenous, and People of Color users than is typically observed in such services. The age distribution also reflected a broad user base, with a median age of 36 years and a mean of approximately 45 years. The accessibility and anonymity of DPS may offer a solution for adults who are underserved by traditional mental health care. These patterns highlight the broader demographic reach of DPS compared with typical services. The flexible, anonymous nature of DPS mitigates certain barriers associated with traditional mental health care, such as time constraints, privacy concerns, and stigma. Users were more likely to engage outside work hours, likely due to an increased sense of psychological safety for vulnerable self-disclosure when outside of work environments. This pattern supports theories of readiness to disclose, which suggest that individuals are more likely to seek help in environments they perceive as emotionally and socially safe [56,57]. Therefore, timing is not only an operational variable but also a sociobehavioral signal of trust, readiness, and psychological safety. Designing digital services that accommodate different levels of readiness would better enable users to engage based on their comfort and needs.

### Discussion Topics

The most commonly discussed topic, by a clear margin, was relationship issues, which appeared in nearly one-third of all live chat sessions. However, differences in topic prevalence emerged when inflows from and outflows to EAP sources were examined. Most frequently, users accessed DPS services through their EAP (inflows) to discuss anxiety, loneliness, physical or mental health, and suicide or self-harm (Table 2). By contrast, users who sought DPS services and were subsequently referred to their EAP programs (outflows) most frequently discussed anxiety, depression, and caregiving or parenting (Table 3). Resource distribution across discussion topics showed that sessions centered on relationship issues received the greatest number of resources, reflecting the high volume of conversations on this topic (Table 4). Trauma, workplace concerns, and stress followed, aligning with their discussion frequency.

### Sentiment Outcomes

Significant improvements in sentiment scores were observed across all investigated sentiment states. The trajectories for loneliness, sadness, stress, anxiety, depression, despair, helplessness, and optimism revealed consistent and statistically significant improvements across all 8 dimensions (Figure 3). Notably, optimism showed the most pronounced change (n=23,491, ~77%; *R*^2^=0.938; *P*<.001), indicating that DPS use was associated with increased feelings of hopefulness and support. The greatest reductions in negative sentiment states were observed in loneliness (n=11,492, 46%; *P*<.001), stress (n=13,928, 46%; *P*<.001), and sadness (n=10,741, 45%; *P*<.001), suggesting that DPS use was associated with nearly halving users’ initial distress.

### Impact of Timing of Use

Consistent with previous research [7], user engagement revealed that nearly two-thirds of sessions occurred outside business hours. This high level of engagement after hours may reflect limited access to traditional in-person or typical tele-mental health support, as well as employees’ greater openness to sharing personal struggles during periods of privacy or reduced work-related stressors.

Users discussed different topics during business hours compared with outside work hours. During business hours, the most common topics were relationship issues, workplace concerns, financial or social determinants of health, trauma, and stress, whereas outside work hours, relationship issues were discussed most frequently, followed by trauma, workplace concerns, stress, and loneliness. Although concerns such as relationship issues were prevalent throughout the day, these differences suggest that users tend to focus more on workplace concerns and social determinants of health during the business day, while outside work hours they are more likely to explore more personal issues such as trauma, stress, and loneliness.

Differences in user activity based on time of use also emerged. Users were more likely to engage for longer periods (Figure 2A), send a higher number of messages and typed characters (Figures 3C and 3D), and discuss more topics (Figure 2E) outside business hours. By contrast, users typed faster during work hours (Figure 2B), suggesting that time constraints related to work responsibilities may limit engagement. Overall, users were able to participate in deeper conversations outside work hours, indicating the need for services that are accessible beyond the typical 9-to-5 window.

Baseline sentiment states differed by time of engagement between the 2 cohorts (Table 5). Users who engaged during business hours reported significantly higher baseline stress, potentially due to situational demands (eg, work or other daytime stressors) [58,59]. By contrast, users who accessed DPS after business hours reported higher baseline levels of depression and loneliness, aligning with research suggesting increased social isolation during evening and nighttime hours, when there are fewer opportunities for social interaction [60,61]. Meanwhile, other sentiment outcomes did not differ by time of day, suggesting that they may be more stable and less influenced by situational factors [62].

These findings suggest 2 distinct sentiment profiles: engagement during business hours is characterized by acute, reactive distress, whereas after-hours engagement reflects more chronic vulnerability. Improvements in sentiment states support these profiles. Depression, helplessness, and loneliness were reduced at higher rates with DPS use outside work hours, suggesting deeper personal engagement (Figure 4). Comparable reductions in anxiety, despair, sadness, stress, and optimism across the 2 groups indicate that these dimensions may be less affected by timing [58-61]. Furthermore, DPS supported psychological relief independent of timing, reinforcing the need for around-the-clock accessibility.

### Impact of In-Session Resource Sharing

Another significant factor influencing sentiment improvements was resource sharing within DPS sessions. Sessions that included at least one resource, such as coping strategies or other psychoeducational materials, were associated with greater positive sentiment changes in anxiety, depression, despair, helplessness, loneliness, stress, and optimism (Figure 5). This finding aligns with the peer support literature, which emphasizes the therapeutic value of shared knowledge and validation of distress [63]. Peer support has been described as “a system of giving and receiving help founded on key principles of respect, shared responsibility, and an agreement of what is helpful” [64]. In digital formats, where users may initially feel disconnected or skeptical, resource sharing may help deepen engagement, enhance perceived value, and reinforce feelings of self-efficacy.

Interestingly, users who did not receive a resource showed slightly greater improvements in sadness, although this difference was minimal. This pattern may suggest that individuals with higher sadness were less likely to seek additional support materials, or that focusing on problem-solving interventions may temporarily heighten awareness of one’s struggles. These nuances highlight the importance of tailoring support strategies to individuals, suggesting that resource sharing may be most impactful for those navigating more acute sentiment states, while others may benefit more from affirmation and increased social connection.

### Limitations and Future Research

While the study provides valuable insights, several limitations should be noted. The observational design prevents causal inference regarding the relationship between session characteristics and sentiment outcomes. An important limitation is that the primary outcome—sentiment change—was based on LLM-derived sentiment scores rather than clinical psychometric instruments. Although prior work and post hoc analyses have shown alignment between LLM-derived sentiment scores and expert human ratings, this study did not examine the psychometric properties of the sentiment scoring approach used. Given the large dataset, human annotation and validation were not conducted. However, post hoc analyses on a subgroup of conversational transcripts confirmed alignment between the model and expert human interpretation. Therefore, the reported sentiment score changes are not diagnostic or absolute measures of emotional change, but rather model-estimated shifts in sentiment within DPS sessions. Despite their relevance in understanding how DPS may support users and potentially encourage engagement with additional clinical care, these results should not be interpreted as indicators of absolute sentiment severity and are not equivalent to self-reported or clinician-scored outcomes. They do not reflect psychiatric symptom severity.

Additionally, limited demographic data restrict analyses of how variables such as age, gender, cultural background, or job role may shape engagement patterns or session efficacy. Although more than half of the users who reported demographic information identified as female and White—a pattern common across digital mental health platforms—DPS attracted a higher proportion of male and Black, Indigenous, and People of Color users than is typically observed. However, the limited demographic data prevented detailed subgroup analyses of sentiment outcomes. Future research should adopt randomized controlled trials or mixed methods designs to identify causal mechanisms underlying DPS effectiveness, while also prioritizing more comprehensive demographic reporting to better capture the service’s impact across diverse populations.

Demographic variables were analyzed for only 8647 out of 24,818 (34.84%) conversations with users who voluntarily disclosed their information. The optional disclosure of personal information introduced selection bias, limiting generalizability to a broader population. A large proportion of individuals may have preferred to withhold information due to privacy, stigma, or other vulnerability-related concerns, and individuals from certain demographic backgrounds may be more likely to face stigma or distrust the health care system, potentially skewing the findings. Therefore, these results may not fully represent the demographic distribution of the broader population; however, they provide valuable insight into user characteristics. The analyzed subset remains sizable and informative regarding the distribution of users in real-world digital mental health settings.

Longitudinal tracking could also reveal whether sentiment improvements persist over time and whether early engagement predicts long-term EAP utilization or mental health outcomes. Moreover, segmenting user data by demographic and occupational variables could clarify subgroup differences, such as among shift workers, caregivers, parents, or underrepresented racial and ethnic groups. Further investigation of digital literacy and privacy preferences as moderating factors could help guide the design of more inclusive digital support systems. Finally, further exploration of resource sharing is warranted. Examining resource types, relevance, and user feedback could help refine the resources provided. Such analyses could also inform best practices for moderator facilitation and content curation within DPS, optimizing its function as a bridge between nonclinical and clinical support systems.

### Practical Implications

The implications of these findings for workplace mental health strategy are substantial. Organizations can benefit from prioritizing awareness-raising efforts for EAP offerings through multimodal, repeated communications, especially during employee onboarding and at moments of organizational stress [54]. Embedding DPS as a core EAP component could expand reach and reduce barriers to support. Leadership endorsement, testimonials, and peer ambassador programs may help reduce stigma and foster a culture of openness [54]. Moreover, training moderators in sentiment attunement and evidence-based resource sharing may enhance user engagement and, in turn, increase the perceived benefits of DPS in improving sentiment state changes [9]. Integrating DPS within EAPs is a scalable approach that could reduce stigma associated with mental health care and improve employee well-being. This approach aligns well with a modern workforce that requires immediate, accessible, and flexible support tools.

### Conclusions

This study underscores the importance of 24/7 access to mental health support, especially for employees who experience emotional distress outside standard business hours. However, because the primary measures were based on LLM-derived sentiment scores, conclusions about effectiveness remain exploratory. Around-the-clock DPS can bridge gaps in care when traditional services are unavailable, helping prevent crisis escalation and demonstrating organizational commitment to employee well-being. To improve EAP utilization, organizations should invest in consistent awareness efforts and destigmatize help-seeking. Leadership endorsement, integration of mental health into everyday communications, and collaboration with employee resource groups can broaden reach and foster a culture of support.

In-session resource sharing demonstrates the value of a hybrid approach that combines AI recommendations with trained peer support moderators to deliver targeted, practical tools. Incorporating psychoeducational resources within sessions may increase user engagement, emotional regulation, and self-efficacy. Furthermore, tailoring resources to users’ expressed needs in real time influences their effectiveness.

Finally, effective mental health strategies must be inclusive and flexible, adapting to diverse schedules, privacy needs, and digital comfort levels. By aligning accessibility with flexibility, employers can offer effective digital mental health interventions to employees. Overall, this study contributes to the growing evidence positioning DPS as an effective, scalable, and equitable tool for mental health across a diverse user base.

## Appendix

Multimedia Appendix 1 Examples provided to interpret emotion intensities in a few-shot manner.

## Abbreviations

AI: artificial intelligence
CCPA: California Consumer Privacy Act
DPS: digital peer support
EAP: employee assistance program
GAD-7: 7-item Generalized Anxiety Disorder
GDPR: General Data Protection Regulation
HIPAA: Health Insurance Portability and Accountability Act
LLM: large language model
NLP: natural language processing
OARS: open-ended questions, affirmations, reflective listening, and summaries
PHQ-9: 9-item Patient Health Questionnaire
PSS-10: 10-item Perceived Stress Scale

## Data Availability

The datasets generated or analyzed during this study are not publicly available due to their proprietary nature, but may be available from Supportiv upon reasonable request. Please contact Supportiv at info@supportiv.com.
