# Supplementary material for: Use of Digital Peer Support for Employee Well-Being: Retrospective Analysis Across Five Large Employers
Source: JMIR Hum Factors. 2026 Apr 7;13:e90431. doi: 10.2196/90431 (PMC13148127; doi:10.2196/90431)
Supplement: Multimedia Appendix 1 [file humanfactors_v13i1e90431_app1.docx]

## Appendix

**Examples provided to interpret emotion intensities in a few-shot manner.**

| 1. **Loneliness**   **Score 1 (low levels of loneliness):**  “I don't share a lot in common with my family, but despite that we still support each other if we can”  **Score 5 (medium levels of loneliness):**  ”My best hope is that I can just find anyone genuine at all. People my age tend to be about as shallow as it gets”  **Score 10 (highest levels of loneliness):**  “I just always feel totally alone. Like no one understands what is going on in my head"   1. **Sadness**   **Score 1 (low levels of sadness):**  “I’m feeling okay today, just want to chat a bit”  **Score 5 (medium levels of sadness):**  ”It's just sad to me that he won't even give me a chance”  **Score 10 (highest levels of sadness):**  “I feel alone and depressed and very dark and invisible. My life has been terrible for years and I never knew what it was like to receive care."   1. **Stress**   **Score 1 (low levels of stress):**  “I feel like I am capable of doing something harder”  **Score 5 (medium levels of stress):**  ”I’ve been feeling a bit overwhelmed with the home renovations lately. It's like every time we finish one thing, another issue pops up. I know we'll get through it, but it's just more stressful than I anticipated.”  **Score 10 (highest levels of stress):**  “I’m so stressed and like my brain is just making me go insane over this like I’m shaking all time just stressed as hell and my mind is racing all the time"   1. **Anxiety**   **Score 1 (low levels of anxiety):**  "I'm feeling okay right now, no major worries."  **Score 5 (medium levels of anxiety):**  ”I'm fairly anxious, often on edge with my stomach in knots, but I'm still getting through the day.”  **Score 10 (highest levels of anxiety):**  “My anxiety and panic attacks are debilitating; I cannot be around people and I have severe insomnia."   1. **Depression**   **Score 1 (low levels of depression):**  “I have ordinary ups and downs, but my mood is generally stable. I enjoy most daily activities and seldom feel discouraged for more than a moment”  **Score 5 (medium levels of depression):**  ”Sadness or emptiness is present most of the day, most days. I still function, yet I must force myself. Interests start to lose their sparkle; concentration and sleep are spotty.”  **Score 10 (highest levels of depression):**  “I feel numb and dissociated and there's nothing I can do about it"   1. **Despair**   **Score 1 (low levels of despair):**  “I'm just gonna try to focus on one day at a time”  **Score 5 (medium levels of despair):**  “I’m trying to push through, but I feel like I'm losing hope”  **Score 10 (highest levels of despair):**  “Just feel like I’m useless to everyone and just a burden”   1. **Helplessness**   **Score 1 (low levels of helplessness):**  “Work was a bit busy today, but overall I feel fine and on top of things”  **Score 5 (medium levels of helplessness):**  “Honestly, I'm struggling. I poured my whole week into that project and it still fell flat. No one even noticed how hard I worked, and I'm feeling really discouraged and in over my head”  **Score 10 (highest levels of helplessness):**  “Life makes me feel sad, spiraling and feel out of control”   1. **Optimism**   **Score 1 (low levels of optimism):**  “Some people are just doomed no matter what they do”  **Score 5 (medium levels of optimism):**  ”Honestly, some days I wonder why I bother, but that's when I am frustrated. I try to imagine the restart”  **Score 10 (highest levels of optimism):**  “It restores and reminds me that there is goodness in people. The warmth and the smile show me humanity is worth every last bit of effort" |
| --- |
